# Case study: Treating infraspinatus and supraspinatus trigger points and supraspinatus tendinopathy utilizing piezoelectric shockwave

**DOI:** 10.3389/fvets.2022.943276

**Published:** 2022-10-26

**Authors:** Heather Owen

**Affiliations:** Animal Acupuncture and Canine Sports Medicine Facility, LLC, Tulsa, OK, United States

**Keywords:** piezoelectric shockwave, musculoskeletal ultrasound, supraspinatus, infraspinatus, myofascial trigger points

## Abstract

Two individual case studies demonstrate piezoelectric shockwave treatment for the resolution of a supraspinatus tendinopathy and supraspinatus and infraspinatus myofascial trigger points (MTPs) *via* musculoskeletal ultrasound. This is the first documentation of improvement of both tendon and muscle fiber patterns in canine patients treated with piezoelectric shockwave. These cases validate the use of piezoelectric shockwave during the rehabilitation of common canine shoulder injuries.

## Introduction

Injuries of the canine shoulder are commonly seen in active large-breed dogs (1–3). The following two cases, with both tendon damage to the supraspinatus muscular tendon junction and myofascial trigger points (MTPs) within the supraspinatus and infraspinatus muscles, demonstrate resolution by utilizing a piezoelectric shockwave. There are previous reports of canine shoulder tendinopathies treated with electrohydraulic shockwave (4, 5). However, these are the first cases with both injuries to the supraspinatus tendon and MTPs in the supraspinatus and infraspinatus muscles treated with piezoelectric shockwave and having ultrasound images showing improved fiber pattern.

Myofascial trigger points are hyper-irritable spots located in a taut band of skeletal muscle at the level of the motor endplate and the sarcoplasmic reticula. They can generate pain and dysfunction and are often caused by mechanical stresses resulting in chronic muscle overload. This results in localized hypoxia and ischemia and the release of inflammatory mediators, which sensitize afferent nerve fibers accounting for the tenderness of the area (6–9). Originally, the only way to visualize a MTP was through MRI. Musculoskeletal ultrasound allows for the visualization of normal and abnormal muscle fiber patterns and is now being used along with palpation skills to identify MTPs (10–14). Utilizing musculoskeletal ultrasound allows for the objective assessment of the resolution of MTPs. Tendinitis can accompany MTPs or can be independent and it occurs most commonly in dogs from repetitive injury or from an acute injury. Musculoskeletal ultrasound has been a way to evaluate tendon structures for fiber damage (10–14). The additional use of musculoskeletal ultrasound to visualize the resolution of MTPs is very useful for the relationship of damaged tissue to pain function of canine patients who cannot communicate *via* human language (15–19).

In human medicine, shockwave had been documented as a useful therapy for both tendon injuries and MTPs. However, in canine patients, shockwave has primarily been used for orthopedic injuries involving bone, tendon, and ligament damage (4, 5, 20–23). Shockwaves are produced by a single pulse high-pressure wave, up to and above 100 MPa. A short rise time and steep slope that occurs in nanoseconds followed by negative pressure, low tensile wave with a small pulse width, both pressure waves occur over about 5–10 μs (24, 25). The Shockwave mechanism of action is well understood as it has been shown to stimulate new blood vessel formation, regulate inflammation, release nitrogen monoxide (NO) that contributes to vasodilation, increase metabolic activity and angiogenesis, and exert an anti-inflammatory effect (26–29). It changes the level of substance P, stimulates bone metabolism, and releases growth factors: IGF, TGF beta, and VEGF gamma (30–34). In addition, shockwave therapy exhibits chondroprotective effects, allows for the dissolution of calcified fibroblasts, stimulates lubricin production, and also stimulates stem cells (35–38).

Shockwaves can be produced using electromagnetic, electrohydraulic, or piezoelectric generators. Electromagnetic and electrohydraulic shockwave modalities are indirectly focused, while piezoelectric shockwaves create direct focused shockwaves. In the cases presented here, direct focused piezoelectric shockwave, PiezoWave^2^-Vet made by Richard Wolf, was chosen as the modality for the treatment of both shoulder tendinopathy and MTPs due to the ability to be precise and deliver pinpoint accuracy of energy. A change in fiber pattern in both the tendon injury and the MTPs was seen *via* musculoskeletal ultrasound after the use of the piezoelectric shockwave and an appropriate rehabilitation plan. This supports *in vitro* and human clinical studies with piezoelectric shockwaves indicating that the therapeutic value of shockwave therapy is independent of the mechanism by which the shockwaves are formed (33, 39–41).

## Case 1: 10-year-old, MN, Great Pyrenees mix

### Case history

The male dog was presented for coxofemoral degenerative joint disease management when pharmaceuticals were not enough to keep him comfortable. He was slow to rise in the morning and was now limping on the right front limb. The dog was slipping on the hardwood floors at home and refusing to go up the stairs. He continued to go for a 1-mile leash walk daily but was unable to get up on furniture and stopped playing. He was taking gabapentin and carprofen prior to being evaluated.

### Initial evaluation

On physical evaluation, the dog had shown decreased hip and shoulder extension. His initial right shoulder extension was 153 degrees, and his left shoulder extension was 153 degrees. He was guarded on right shoulder extension and had MTPs in the right supraspinatus and infraspinatus muscles. There was pain on supraspinatus tendon palpation. Body condition score was seven out of nine and a lameness score of 3/5 RF (42). The pain score was 2/4 according to the Colorado pain score (42, 43). Digital thermography confirmed the physical evaluation findings. Shoulder radiographs were normal.

**Figure 1:**
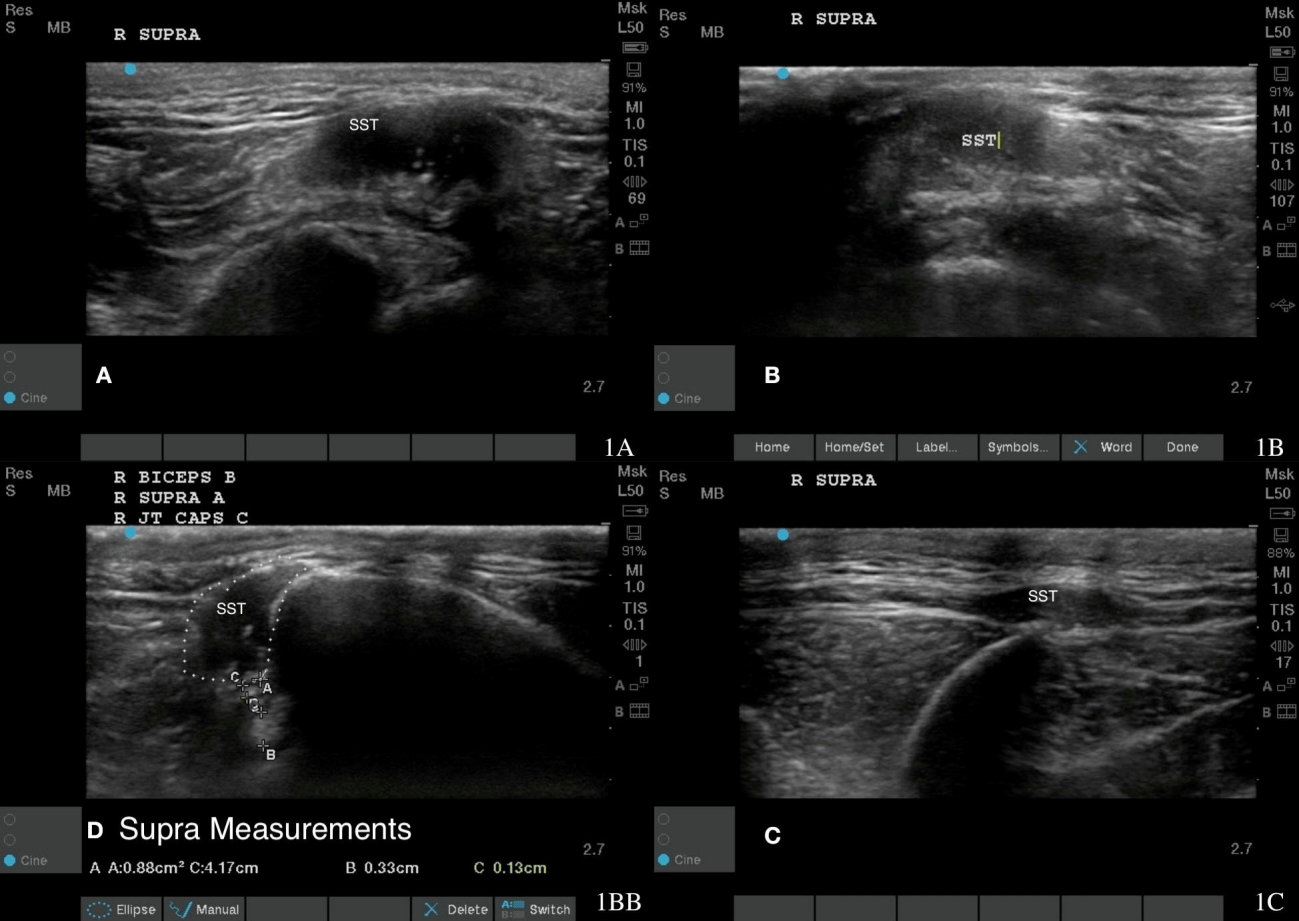
Case 1-Right supraspinatus insertional tendinopathy. **(A)** Original image revealing hyperechoic mixed echogenicity within supraspinatus tendon. **(B)** More homogenous tendon fiber pattern is shown at 8 weeks post shockwave therapy. **(C)** A normal fiber pattern is shown at 18 months post shockwave therapy. **(D)** revealed supra measurement at 18 months post shockwave therapy.

**Figure 2:**
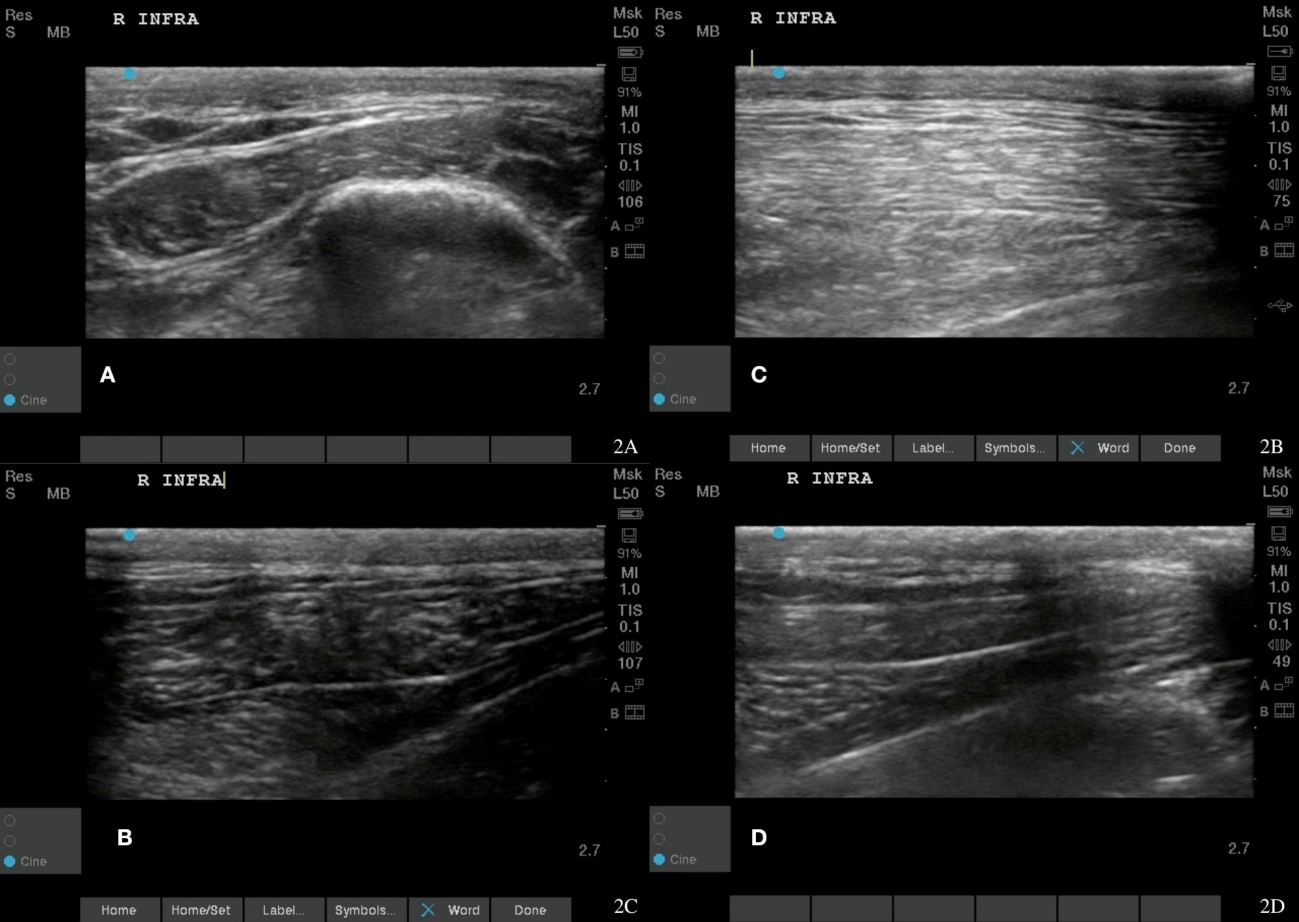
Case 1-Right infraspinatus myofascial trigger points (MTPs). **(A)** Original image revealing irregular muscle fiber pattern with hyperechoic and wavy fibers indicating fiber pattern disruption. **(B)** A normalizing muscle fiber pattern is shown at 8 weeks post shockwave therapy. **(C,D)** The normal tendon and muscle fiber patterns are shown at 18 months post shockwave therapy.

**Figure 3:**
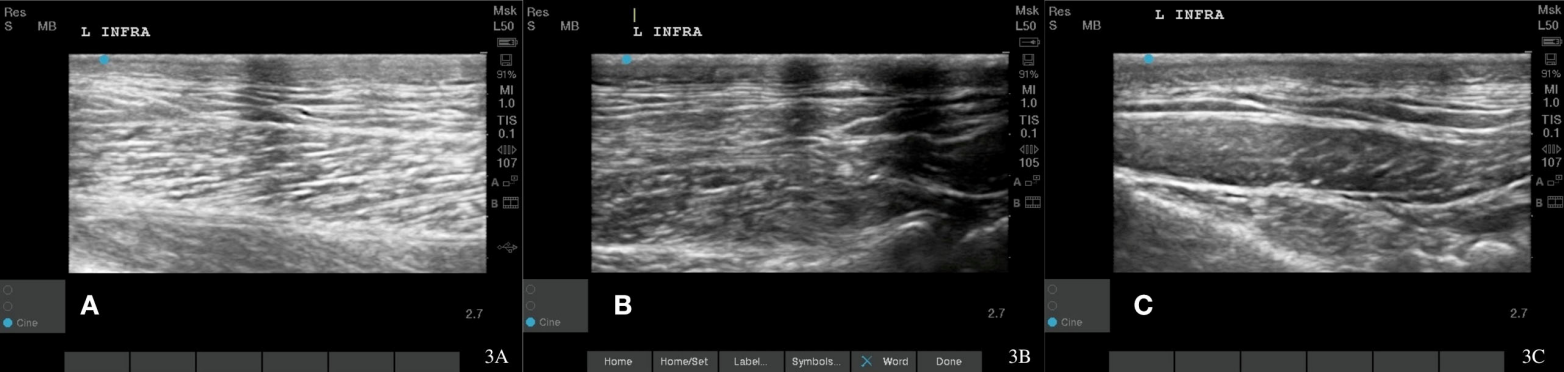
Case 1-Left supraspinatus MTP. **(A)** Original image revealing muscle fiber pattern disruption with hypoechoic areas throughout the muscle. **(B)** The wavy muscle fiber patterns with less hypoechoic areas is shown at 8 weeks post shockwave therapy. **(C)** The normal tendon and muscle fiber patterns are shown at 18 months post shockwave therapy.

**Figure 4:**
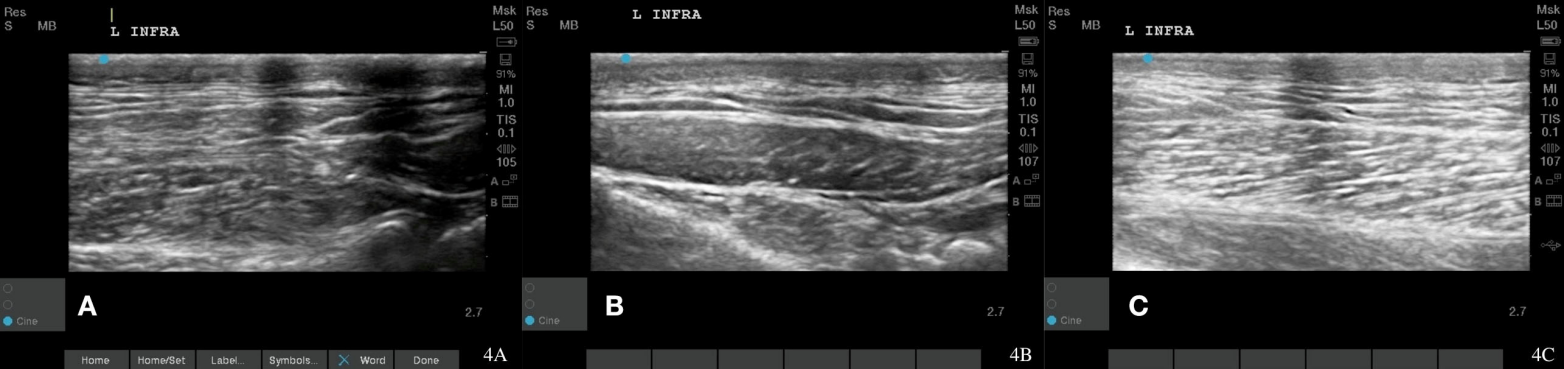
Case 1-left infraspinatus MTP. **(A)** Original image revealing irregular muscle fiber pattern. **(B)** A normalizing muscle and tendon fiber pattern is shown at 8 weeks post shockwave therapy. **(C)** Normal muscle and tendon fiber patterns are shown at 18 Month post shockwave reveals.

Musculoskeletal ultrasound images were obtained prior to any treatment being administered. Left supraspinatus fibers near the musculotendinous junction had an irregular fiber pattern, supraspinatus insertional tendinopathy in the right shoulder (Figure 1A). There were also hypoechoic areas and fiber pattern disruption in both the right infraspinatus and left supraspinatus muscles (Figures 2A, 3A). Bilateral infraspinatus and supraspinatus myofascial trigger points were identified using musculoskeletal ultrasound (Figures 2A, 3A, 4A).

### Choice of therapy

A piezoelectric shockwave was utilized to treat the MTPs present in the supraspinatus and infraspinatus muscle groups in addition to the inflammation of the right supraspinatus tendon. A 15 mm stand-off pad was utilized and a frequency of eight shocks/s for a total of 1,000 shocks at 0.032 mj/mm^2^ for the trigger point and at 0.039 mj/mm^2^ for the supraspinatus tendon. Two treatments were needed for the trigger point and four treatments were needed to resolve the tendonitis. After four treatments with the piezoelectric shockwave, the musculoskeletal ultrasound revealed tendon healing and MTP resolution (Figures 1B, 2B, 3B). The supraspinatus and biceps tendon were measured in cross section (Figure 1D). The muscles and tendons were reimaged at 18 weeks post treatment (Figures 2D, 3C). The dog's pain scale decreased to a one out of four on the Colorado pain score and rehabilitation was started to decrease lameness, increase function, and increased range of motion to the shoulder and coxofemoral joints. Land rehabilitation was implemented to strengthen the affected muscles and to address the compensation. In total, four shockwave treatments were performed. Rehabilitation is ongoing at monthly maintenance intervals due to the chronic degenerative joint disease of the coxofemoral joints.

### Outcome

Rechecks of the dog were performed at 2 weeks and then every 4 weeks until 18 months post treatment. These involved pain assessment, gait analysis, stance analysis, goniometry, Gulick tape measurements, myofascial palpation, digital thermography, and musculoskeletal ultrasounds. Musculoskeletal ultrasound of the supraspinatus tendon and supraspinatus and infraspinatus muscles 8 weeks after starting shockwave therapy revealed a normal fiber pattern of the infraspinatus and supraspinatus muscles, normal echogenicity of the supraspinatus tendon, and a decrease in the overall size of the supraspinatus tendon (Figures 1B,C, 2B,C, 3B,C). At this time, the dog had a lameness score of 1/5 RF. No pain on supraspinatus tendon palpation and right shoulder extension had risen to near normal. The left shoulder extension had increased to 159 degrees and the right shoulder extension had increased to 161 degrees. To date, this dog is 0/5 lame, remains 0/4 on the Colorado pain score, and the owner describes him as back to “acting like a puppy.” He is able to run, jump, climb stairs, go for walks, and get up on furniture again. By incorporating a home exercise program, disease-modifying nutraceuticals, and anti-slip flooring and maintenance rehabilitation, this dog has not had any further pain or dysfunction.

## Case 2: 9-year-old, male neutered Labrador Retriever

### Case history

The male dog presented for limping on his left front limb after he was found at the bottom of a drainage ditch, and was unable to come out. He had tibial-plateau-leveling osteotomy (TPLO) of his left stifle at 5 years of age and TPLO of his right stifle at 2 years of age. He has geriatric onset laryngeal paralysis polyneuropathy, laryngeal paralysis, right stifle degenerative joint disease, hyperadrenocorticism, and hypothyroidism. The dog was currently taking carprofen, gabapentin, adequan, and on disease-modifying neutraceuticals.

### Initial evaluation

**Figure 5:**
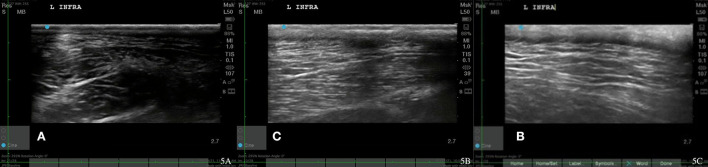
Case 2-Left infraspinatus MTP. **(A)** Original injury revealing hypoechoic areas and irregular muscle fiber patterns. **(B)** Myofascial trigger points resolution, 1-week post injury after 2nd shockwave therapy reveals normalizing muscle. **(C)** Follow up, 2 weeks after MTP resolution with normal muscle fiber pattern.

On physical evaluation, the dog had a large MTP near the tendon of insertion on his left infraspinatus. He had pain with full left shoulder extension and full flexion. He had crepitus in his left stifle and mild muscle guarding on stifle extension and left and right tarsal valgus secondary to TPLO procedures. His left stifle extension was 144 degrees, and his right stifle extension was 133 degrees. Goniometry was not recorded for his shoulders at this time. His body condition score was seven out of nine and a lameness score of 2/5 LF (42). The pain score was 2/4 according to the Colorado pain score (42, 43). The dog also has mild conscious proprioception deficits in hind limbs due to geriatric onset laryngeal paralysis polyneuropathy. Shoulder radiographs were normal. Musculoskeletal ultrasound images were obtained prior to any treatment administered. Left infraspinatus fibers had an irregular fiber pattern (Figure 5A). Musculoskeletal ultrasound diagnosis was infraspinatus MTPs.

### Choice of therapy

The plan for treatment involved four shockwave treatments over the infraspinatus muscle. 145 Piezoelectric shockwave, the Piezowave2-Vet made by Richard Wolf, was utilized to treat the MTP in the left infraspinatus muscle group. A 15 mm linear stand-off pad and a frequency of eight shocks/s for a total of 1,000 shocks at 0.046 mJ/mm^2^ were used. In total, four piezoelectric shockwave treatments were performed at a 2x/week interval.

### Outcome

After 4 treatments, the musculoskeletal ultrasound revealed the resolution of the MTP (Figure 5B). The patient's pain scale decreased to a score of 1/4 on the Colorado pain score and rehabilitation was started to decrease lameness, increase function, and increase the range of motion to the left-shoulder joint.

After the resolution of the trigger point, land rehabilitation was implemented. Land rehabilitation was utilized to strengthen the affected muscles and address the compensation and address decrease in the range of motion and aid in flexibility and proprioception. Rehabilitation is ongoing at monthly maintenance intervals due to the chronic degenerative joint disease of the left and right stifle joints and concurrent geriatric onset laryngeal paralysis polyneuropathy.

Recheck musculoskeletal ultrasound of the infraspinatus 8 and 12 weeks after starting shockwave therapy revealed a normal fiber pattern of the infraspinatus and supraspinatus muscles (Figures 3C, 4B,C, 5B,C). Lameness score of 1/5 on right hind remains, and patient resolved lameness on the left front limb. No pain in infraspinatus muscle or tendon palpation or shoulder range of motion. To date, this patient remains 0/4 on the Colorado pain score. The owner describes him as “back to his young self.” He continues to run, jump, climb stairs, go for walks, and get up on furniture. He is back to running in the drainage ditches and helping to keep the property free from wildlife. By incorporating a home exercise program, anti-slip flooring, and maintenance rehabilitation, this patient has not had any further pain or dysfunction.

## Conclusion

Muscle sprains, tendinopathies, and MTPs are common in practice. How we manage and treat these common occurrences is ever evolving. Incorporating shockwave early in the treatment of these conditions resulted in quicker resolution of pain, faster resolution of lameness and discomfort for the dog, and increased function of the muscles and tendons (3, 4, 7, 20–23, 34, 36). While palpation of the myofascial structures can never be underestimated, being able to “see” the healing with the use of digital thermography and musculoskeletal ultrasound help to give us a more objective analysis of the resolution utilizing different modalities, including piezoelectric shockwave, therapeutic ultrasound, and regenerative medicine, as we work together to further understand how to better treat our patients (10–14, 16, 42).

The piezoelectric shockwave mechanism of action is well understood and used regularly for human therapy medicine (33, 38–41). However, due to being a site specific direct focused shockwave, its efficacy has been questioned in veterinary medicine. MTPs allow for the perfect place to start with evidence to assess how piezoelectric shockwave can be utilized in veterinary medicine. With the understanding that shockwave can be an important part of tendon healing, ligament healing, and osteoarthritis management in animals, more information is needed to evaluate its effect on myofascial trigger points.

With the addition of piezoelectric shockwave and rehabilitation exercises, the following patients were able to keep their muscle and musculocutaneous junction intact and are maintaining function. Historically, it has been documented that tendon damage and MTPs respond to conservative management with manual trigger point release, shockwave, regenerative medicine, rest, and corticosteroid injection into the bursa or tendon (4, 7, 20, 44). However, some patients do require surgical resection and release of the tendon.

These case reports are initial documentation of the ability of piezoelectric shockwave to heal both Canine tendon and muscle injuries containing diagnostic ultrasound images. Tendon injuries are often documented with musculoskeletal ultrasound; however, especially in veterinary medicine, ultrasound images of trigger points and other muscular damage are not as widely available. Advances in musculoskeletal ultrasound techniques are allowing veterinarians to equally image both tendon and muscle fibers to help better determine when appropriate rehabilitation exercise should be implemented.

There are clear limitations in drawing significant conclusions from just two retrospective cases. Prospective, controlled, and clinical studies are needed to make a full comparison of piezo extracorporeal shockwave therapy (ESWT) technology to electrohydraulic technology. However, it is important to recognize there is initial objective data in veterinary medicine supporting the hypothesis that the means by which the shockwave is formed does not have a direct effect on the biological response of the tissue. The appropriate amount of energy and the path of the energy is very important. However, dosing for therapeutic shockwave is a difficult topic that needs to be better understood.

## Data availability statement

The original contributions presented in the study are included in the article/supplementary material, further inquiries can be directed to the corresponding author/s.

## Ethics statement

Ethical review and approval was not required for the animal study because no animal was sedated or injured during these case reports. Owners gave permission to treat. Written informed consent was obtained from the owners for the participation of their animals in this study.

## Author contributions

The author confirms being the sole contributor of this work and has approved it for publication.

## Conflict of interest

Author HO was employed by the company Animal Acupuncture and Canine Sports Medicine Facility, LLC.

## Publisher's note

All claims expressed in this article are solely those of the authors and do not necessarily represent those of their affiliated organizations, or those of the publisher, the editors and the reviewers. Any product that may be evaluated in this article, or claim that may be made by its manufacturer, is not guaranteed or endorsed by the publisher.
